# Partial cross-protection between Japanese encephalitis virus genotype I and III in mice

**DOI:** 10.1371/journal.pntd.0007601

**Published:** 2019-08-02

**Authors:** Jianchao Wei, Xin Wang, Junjie Zhang, Shuang Guo, Linlin Pang, Kun Shi, Ke Liu, Donghua Shao, Yafeng Qiu, Lihong Liu, Frederik Widén, Beibei Li, Zhiyong Ma

**Affiliations:** 1 Department of Swine Infectious Diseases, Shanghai Veterinary Research Institute, Chinese Academy of Agricultural Science, Shanghai, PR China; 2 Department of Virology, Immunobiology and Parasitology (VIP), The National Veterinary Institute (SVA), Uppsala, Sweden; Oxford University Clinical Research Unit, VIET NAM

## Abstract

Genotype III (GIII) Japanese encephalitis virus (JEV) predominance has gradually been replaced by genotype I (GI) over the last 20 years in many Asian countries. This genotype shift raises concerns about the protective efficacy of Japanese encephalitis (JE) vaccines, as all of the currently licensed JE vaccines are derived from GIII strains. In this study, we conducted vaccination-challenge protection assays to evaluate the cross-protective efficacy of GI- or GIII-derived vaccines against the challenge of a heterologous genotype using a mouse challenge model. Titration of the neutralizing antibodies elicited by SA14-14-2 live-attenuated JE vaccine (SA14-14-2 vaccine), a GIII-derived vaccine, indicated that the titer of neutralizing antibodies specific to heterologous genotype GI stain was significantly lower than that specific to homologous genotype GIII strain in both pigs and mice immunized with the SA14-14-2 vaccine. Vaccination of mice with SA14-14-2 vaccine or a GIII-inactivated vaccine at high and medium doses completely protected vaccinated mice against challenge with the homologous genotype GIII strains, but failed to provide the vaccinated mice complete protection against the challenge of heterologous genotype GI strains. The protection rates against GI strain challenge were 60%–80%, showing that these vaccines were partially protective against GI strain challenge. Additionally, vaccination of mice with a GI-inactivated vaccine conferred 100% protection against the challenge of homologous genotype GI strains, but 50%–90% protection against the challenge of heterologous genotype GIII strains, showing a reduced protective efficacy of a GI-derived vaccine against GIII strain challenge. Overall, these observations demonstrated a partial cross-protection between GI and GIII strains and suggested a potential need for new JE vaccine strategies, including options like a bivalent vaccine, to control both genotype infection.

## Author summary

Japanese encephalitis virus (JEV) is a mosquito-borne flavivirus that causes Japanese encephalitis (JE) in humans and reproductive disorders in pigs. JEV is phylogenetically classified into five genotypes. JEV genotype III (GIII) was historically dominant throughout most of Asia, but has been replaced by genotype I (GI) over the last 20 years in many Asian countries. Amino acid variations in JEV envelope protein play major roles in determination of antigenicity. Elicitation of cross-neutralizing antibodies for GI and GIII strains has been reported, showing an antigenic difference between the two genotypes. These amino acid differences in JEV envelope proteins raise a concern about the protective efficacy of JE vaccines against the emerged GI strain infection, because all currently licensed JE vaccines are derived from GIII strains. We evaluated the protective efficacy of JE vaccines against the heterologous genotype strain using a mouse challenge model and found a partial cross-protection between GI- or GIII-derived vaccines against the challenge of the heterologous genotype. This partial cross-protective efficacy suggested a potential need for a new JE vaccine, one solution may be a bivalent vaccine, to control infection with either genotype. However, more comprehensive studies should be conducted to address the partial cross-protective efficacy of JE vaccines against the heterologous genotype strains using JEV natural hosts such as pigs.

## Introduction

Japanese encephalitis virus (JEV) is the causative agent of Japanese encephalitis (JE) which is prevalent in ~25 Asia-Pacific countries, with the estimated number of human cases ranging from 50,000 to 175,000 each year [1]. Globally, approximately 75% of human cases happen in children and adolescents, making JE the leading cause of viral childhood encephalitis in Asia [2]. In addition to humans, pigs and horses are susceptible to JEV infection. Infection with JEV causes abortion in pregnant sows and encephalitis in piglets and horses [3], which results in JEV being a significant public health and economic risk.

JEV is a mosquito-borne flavivirus with a single-stranded positive-sense RNA genome that encodes three structural and seven nonstructural proteins. Although JEV exists as a single serotype, it is phylogenetically classified into five genotypes (genotype I to V) based on the nucleotide sequence of the viral envelope (E) protein [4,5]. JEV genotype III (GIII) was the historically dominant genotype throughout most of Asia but has been gradually replaced by genotype I (GI) over the last 20 years in many Asian countries. Surveillance studies have been able to demonstrate this genotype shift [6,7]. Although GI has replaced GIII as the dominant genotype, co-circulation of GI and GIII viruses is present in many Asian countries [8–10].

Vaccination is the most effective way to control JE in both humans and pigs [3,11]. However, all currently licensed JE vaccines including the widely used SA14-14-2 live-attenuated JE vaccine (SA14-14-2 vaccine) that is one of JE vaccines recommended by the World Health Organization are derived from GIII strains [12]. The emergence of the GI strain as the dominant genotype has raised concerns about the effectiveness of GIII-derived vaccines against the GI strain infection [13].

JEV E protein is the major structural protein containing the receptor-binding domain and the neutralization epitopes. This protein plays major roles in determination of antigenicity and elicitation of neutralizing antibodies [14,15]. Mutations in the E protein influence cell tropism, virulence, and antigenicity of JEV [15–17]. Amino acid variations in the E protein have been reported between GI and GIII strains [10,18,19], showing an antigenic difference between the two genotypes [20]. Reduced levels of neutralizing antibodies against GI strains have been observed in both humans and pigs vaccinated with GIII-derived vaccines [18,21]. In addition, GI viruses have been isolated from JE patients vaccinated with the SA14-14-2 vaccine [19,22]. These previous observations imply a reduced protective efficacy of GIII-derived vaccines against GI strain infection. In this study, we conducted vaccination-challenge protection assays using a mouse challenge model to evaluate the cross-protective efficacy of GI- and GIII-derived vaccines against heterologous genotype infection.

## Materials and methods

### Ethics statement

All animal experiments were approved by the Institutional Animal Care and Use Committee of Shanghai Veterinary Research Institute, China (IACUC No: Shvri-mo-2017091601) and performed in compliance with the Guidelines on the Humane Treatment of Laboratory Animals (Ministry of Science and Technology of the People’s Republic of China, Policy No. 2006 398).

### Virus and cells

JEV strains including two GI strains (SD12 and SH7), four GIII strains (N28, SH1, SH15 and SH19) [23] and the SA14-14-2 vaccine strain (GenBank No. AF315119) were used in this study. All JEV strains were grown and tittered on newborn hamster kidney cells (BHK-21), which were maintained in Dulbecco’s modified Eagle’s medium (DMEM; Thermo Fisher Scientific, Carlsbad, CA, USA) supplemented with 10% fetal bovine serum (FBS) at 37°C in an atmosphere containing 5% CO_2_. After inoculation with JEV, BHK-21 were cultured in DMEM supplemented with 2% FBS at 37°C. 50% lethal dose (LD50) of each JEV strain was tested on three-week-old C57BL/6 strain mice by intraperitoneal inoculation of serially diluted JEV (S1 Table).

### Multiple amino acid sequence alignment

Amino acid sequences of JEV strains were obtained from GenBank (S1 Table). Multiple sequence alignments on amino acid sequence of JEV E protein were performed using the DNASTAR Lasergene 7.1 (MegAlign).

### Preparation of inactivated vaccines

JEV was inactivated with binary ethylenimine (BEI) as described previously [24,25]. Briefly, GI SD12 strain (SD12(GI)) and GIII N28 strain (N28(GIII)) were cultured in BHK-21 cells and harvested at 3 days post-inoculation with 80% cytopathogenic effect. Following three cycles of freeze-thaw, the supernatants were collected by centrifugation and tittered on BHK-21 cells. The tittered viral supernatants were treated with BEI at final concentration of 0.1 mM for 10 h at 37°C and the inactivation was stopped with 2 mM sodium thiosulfate. Inactivation was verified by inoculation of the BEI-inactivated viruses on BHK-21 cells for 7 days. The inactivated virus was emulsified with an equal volume of ISA206 adjuvant (Seppic, Paris, France). One dose of the inactivated vaccine contained a viral titer of 10^5^ plaque-forming units (PFU; 0.3 ml/each).

### Examination of vaccine protective efficacy in mice

Four-week-old C57BL/6 strain mice purchased from the Shanghai SLAC Laboratory Animal Co. LTD were randomly divided into the vaccinated and control groups (*n* = 10 mice/group). Vaccination and challenge were performed as previously described [26]. For vaccination with the SA14-14-2 vaccine, mice were intraperitoneally injected with SA14-14-2 vaccine (Lot. 201706116, Wuhan Keqian Biology, Wuhan, China) at a dose of 5000, 500, or 50 PFU per animal and challenged intraperitoneally with a dose of 20 LD_50_ of JEV at 14 days post-vaccination. For vaccination with the inactivated JE vaccine, mice were intraperitoneally injected with N28(GIII)- or SD21(GI)-inactivated vaccine at a dose of 10^5^,10^4^, or 10^3^ PFU per animal and boosted with the same dose at 14 days post-primary vaccination. The mice were challenged intraperitoneally with a dose of 20 LD_50_ of JEV at 14 days post-vaccine boost. The challenged mice were monitored daily for 20 days and the mortality rates were calculated accordingly.

### Immunization of animals with SA14-14-2 vaccine

Six-month-old clinically healthy and JEV antibody negative crossbred pigs (*n* = 40) were intramuscularly immunized with SA14-14-2 vaccine at a dose of 10^5^ PFU per animal. Serum samples from each animal was collected at 30 days post-vaccination for detection and titration of neutralizing antibodies. Four-week-old C57BL/6 strain mice (*n* = 10) were intramuscularly injected with the SA14-14-2 vaccine at a dose of 10^5^ PFU per animal and serum samples were collected at 14 days post-vaccination for detection of neutralizing antibody titers.

### Detection of neutralizing antibody titers

Neutralizing antibodies in serum samples collected from vaccinated animals were tittered using the plaque reduction neutralization test (PRNT), as described previously [18]. Briefly, serum samples were inactivated in a water-bath for 30 min at 56°C and serially diluted two-fold. The diluted serum samples (0.1 ml) were mixed with an equal volume of JEV at a concentration of 200 PFU/0.1 ml and incubated for 1 h at 37°C. The mixture was subsequently dispensed onto BHK-21 cells grown in 6-well plates and incubated for 2 h at 37°C. After 2 h adsorption, the cells were overlaid with 1.2% methylcellulose (Thermo Fisher Scientific) in DMEM containing 2% FBS and incubated for 3−5 days at 37°C. The plaques were stained with crystal violet and counted. Neutralizing antibody (PRNT_50_) titers were calculated as the reciprocal of the highest dilution that reduced the plaque numbers by at least 50% relative to the virus control. The PRNT_90_ titers that reduced the plaque numbers by at least 90% relative to the virus control were also calculated. The positive cut-off value of neutralizing antibody titers was defined as PRNT_50_ ≥ 10. PRNT_50_ titers below the positive cut-off value of 10 were given an arbitrary value of 5 for the calculation of the geometric mean titer (GMT) [18].

### Statistical analysis

All data were processed using GraphPad Prism 7.0 (GraphPad, La Jolla, CA, USA). Fisher’s exact test or Student’s *t*-tests were used for statistical analyses. A *p* value < 0.05 was considered statistically significant.

## Results

### Amino acid variations in the E protein between GI and GIII strains

We compared the amino acid differences of the E protein among GI and GIII strains used in this study. A total of nine amino acid variations were detected in the E protein between the strains, of which four, highlighted in red, were conserved between the GI and GIII strains including the SA14-14-2 vaccine strain. The varied residues of T129M and A222S are located in domain II (DII), and S327T and A366S are present in domain III (DIII) of the E protein (Table 1). DIII plays a crucial role in elicitation of neutralizing antibodies [14], therefore the variations of S327T and A366S present in DIII may alter the cross-protection of the SA14-14-2 vaccine against GI strain infection.

**Table 1:** Amino acid variations in E protein among JEV strains.

Strain	Genotype	Amino acid residues								
		DI	DII						DIII	
		176	123	129	138	209	222	244	327	366
**SA14-14-2**	III	V	S	T	K	K	A	G	S	A
**N28**	III	I	R	T	E	R	A	E	S	A
**SH1**	III	T	R	T	E	K	A	E	S	A
**SH15**	III	T	R	T	E	K	A	G	S	A
**SH19**	III	I	R	T	E	R	A	E	S	A
**SD12**	I	I	S	M	E	K	S	E	T	S
**SH7**	I	I	S	M	E	K	S	E	T	S

### Low PRNT_50_ titers of neutralizing antibodies specific to heterologous genotype SD12(GI) strain in vaccinated pigs and mice

Given the amino acid variations in the E protein between GI and GIII strains (Table 1), we measured the neutralizing antibody titers elicited by the SA14-14-2 vaccine against the heterologous genotype SD12(GI) strain. Serum samples were collected from pigs (*n* = 40) vaccinated with SA14-14-2 vaccine and the neutralizing antibody titers (PRNT_50_) specific to the homologous SA14-14-2, homologous genotype N28(GIII) and heterologous genotype SD12(GI) strains were measured, respectively.

**Fig 1:**
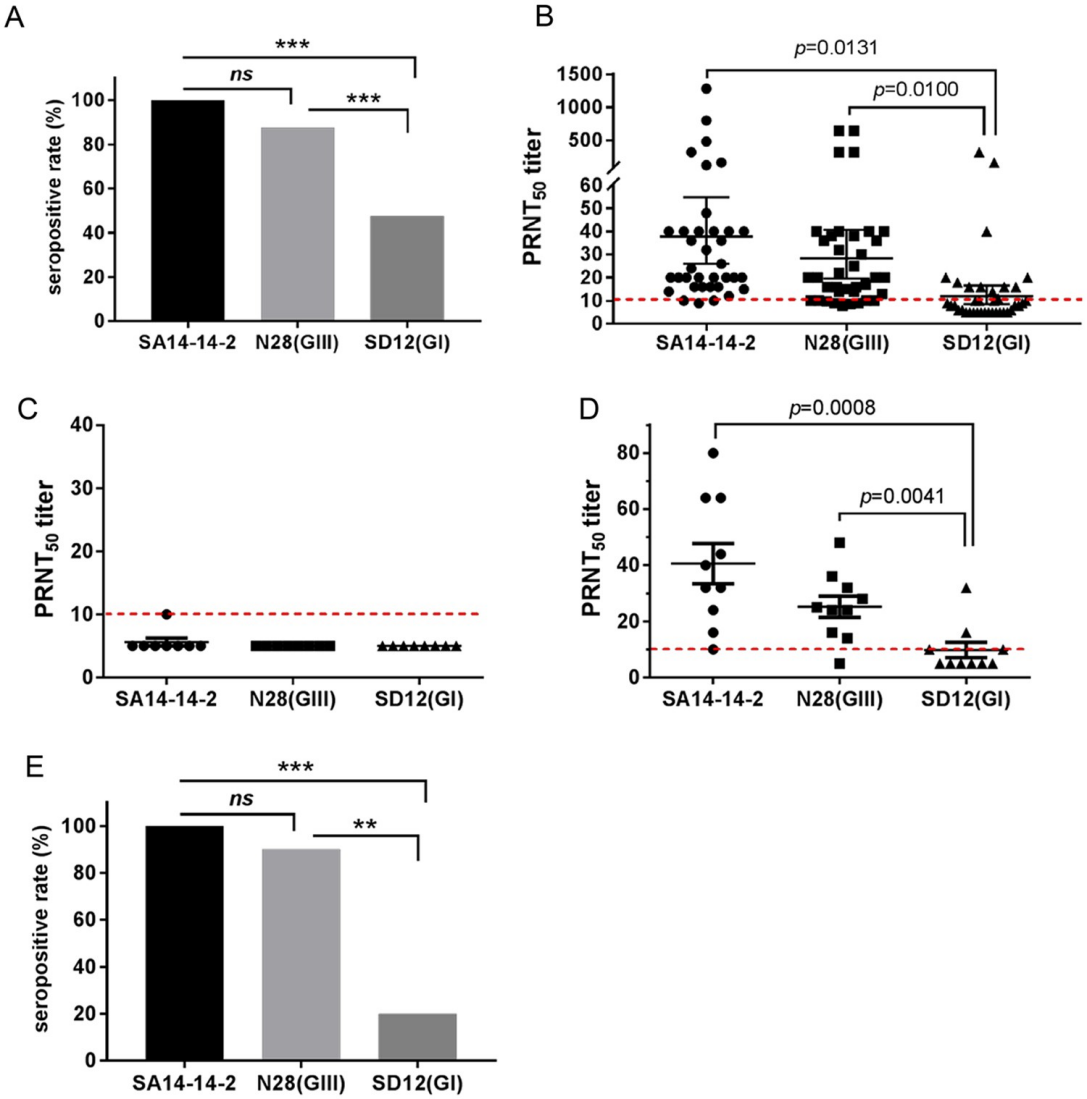
Detection of neutralizing antibody titers in pigs and mice vaccinated with the SA14-14-2 vaccine. (A and B) Pigs (*n* = 40) were immunized with SA14-14-2 vaccine and serum samples for each animal were collected at 30 days post-vaccination for detection of neutralizing antibodies against SA14-14-2, N28(GIII) and SD12(GI) strains. (A) Seropositive rate against the indicated JEV strains. ***, *p* < 0.001 detected by Fisher’s exact test. (B) Neutralizing antibody titers (PRNT_50_) against the indicated JEV strains were measured on BHK-21 cells and plotted. A *p* value was generated by Student’s *t*-test. (C, D and E) Mice (*n* = 10) were immunized with the SA14-14-2 vaccine and serum samples were collected at 14 days post-vaccination for detection of neutralizing antibodies against SA14-14-2 vaccine, N28(GIII) and SD12(GI) strains. (C) PRNT_50_ titers in serum samples collected from pre-vaccinated mice. (D) PRNT_50_ titers against the indicated JEV strains in serum samples collected from vaccinated mice. A *p* value was generated by Student’s *t*-test. (E) Seropositive rate against the indicated JEV strains. ***, *p* < 0.001, **, *p* < 0.01 detected by Fisher’s exact test.

The seropositive rate, as defined by PRNT_50_ ≥10, against the homologous SA14-14-2 strain was 100% (40/40), showing a similar trend to that (87.5%, 35/40) of the homologous genotype N28(GIII) strain. However, the seropositive rate against the heterologous genotype SD12(GI) strain was 47.5% (19/40) which was significantly lower than those of the homologous SA14-14-2 (*p*<0.0001) and homologous genotype N28(GIII) (*p* = 0.0003) strains (Fig 1A).

The PRNT_50_ titer against the homologous SA14-14-2 strain was 38.0, which was close to that (23.5) of the antibody specificity to the homologous genotype N28(GIII) strain, but significantly higher than the antibody specificity (8.8) for the heterologous genotype SD12(GI) strain (Fig 1B). The serum samples were stratified into different groups of PRNT_50_ titer 10, 20, 40, 80, 160 and ≥ 320 specific to the homologous SA14-14-2 strain, as described previously [18]. The GMT of the specific PRNT_50_ titers to the homologous genotype N28(GIII) strain and the heterologous genotype SD12(GI) strain were calculated for each group. The cross-protective threshold (PRNT_50_ = 10) to the homologous genotype N28(GIII) strain and the heterologous genotype SD12(GI) strain was approximately equivalent to the PRNT_50_ titer of 10 and 40 against the homologous SA14-14-2 strain, respectively (Table 2). These data indicated a low presumptive protective titer of neutralizing antibodies against the heterologous genotype SD12(GI) strain in pigs vaccinated with the SA14-14-2 vaccine.

**Table 2:** Geometric mean titers (GMT) of PRNT50 titers specific to N28(GIII) and SD12(GI) strains in vaccinated pigs.

PRNT_50_ against SA14-14-2	Serum samples from vaccinated pigs		
	Sample size	PRNT_50_ GMT against JEV (95% CI)	
		N28(GIII)	SD12(GI)
**10**	7	9.2	5.1
**20**	10	16.3	7.1
**40**	8	22.9	10.5
**80**	6	35.3	12.8
**160**	5	64.0	30.2
**≥ 320**	4	480.0	160.0

To confirm this observation, mice (*n* = 10) were vaccinated with the SA14-14-2 vaccine and the neutralizing antibody titers against the homologous SA14-14-2, homologous genotype N28(GIII) and heterologous genotype SD12(GI) strains were measured. Serum samples collected from pre-vaccinated mice showed a background level of PRNT_50_ titers to almost of JEV strains, with one exception that showed the PRNT_50_ titer of 10 (Fig 1C). In response to vaccination, the PRNT_50_ titer against the homologous SA14-14-2 strain reached 40.6 above the background. The PRNT_50_ titer specific to the homologous genotype N28(GIII) strain was 25.2 which was lower than that produced against the SA14-14-2 strain, but higher than that (9.8) against the heterologous genotype SD12(GI) strain (Fig 1D). The seropositive rate against the homologous SA14-14-2 strain was 100% (10/10), showing a similar trend to that (90%, 9/10) of the homologous genotype N28(GIII) strain. However, the seropositive rate against the heterologous genotype SD12(GI) strain was only 20% (2/10) which was significantly lower than the vaccine strain (*p* = 0.0007) or the homologous genotype N28(GIII) (*p* = 0.0055) strain (Fig 1E). Additionally, PRNT_90_ assay was used to detect neutralizing antibodies against the homologous and heterologous genotype strains. A reduced neutralizing antibody titer against the heterologous genotype was also observed (S1 Fig). Taken together these data support the hypothesis of reduced neutralizing antibody response against the heterologous genotype following vaccination.

### Partial protective efficacy of SA14-14-2 vaccine against heterologous genotype SD12(GI) strain challenge in mice

**Fig 2:**
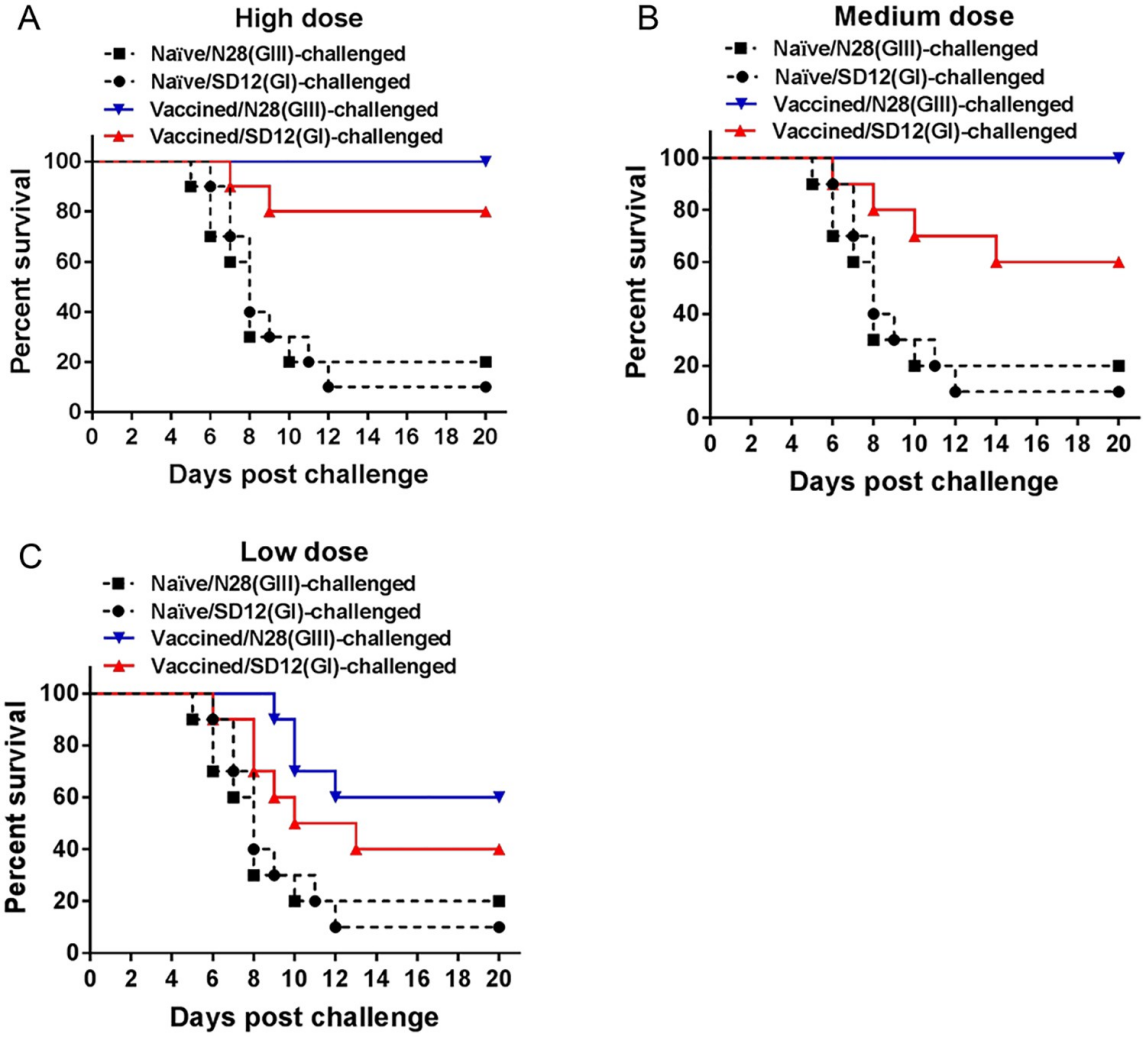
Protective efficacy of the SA14-14-2 vaccine. Mice (*n* = 10) were mock-vaccinated (Naïve) or immunized with SA14-14-2 vaccine at high (5000 PFU) dose (A), medium (500 PFU) dose (B), and low (50 PFU) dose and challenged with the indicated JEV strains.

Given the low PRNT_50_ titers of neutralizing antibodies specific to the heterologous genotype SD12(GI) strain in animals vaccinated with SA14-14-2 vaccine, we tested the protective efficacy of SA14-14-2 vaccine against SD12(GI) strain challenge. Pigs excluding pregnant sows are generally subclinical after JEV infection [27]. We therefore used mice that are susceptible to JEV infection to evaluate the protective efficacy of the SA14-14-2 vaccine against SD12(GI) strain challenge. Mice were vaccinated with the SA14-14-2 vaccine at high (5000 PFU), medium (500 PFU) or low (50 PFU) doses, respectively, and challenged with the homologous genotype N28(GIII) strain and the heterologous genotype SD12(GI) strain. The SA14-14-2 vaccine completely protected vaccinated mice at the high and medium doses, but not at the low dose (percent survival = 60%), against challenge with the homologous genotype N28(GIII) strain (Fig 2). However, the vaccine failed to provide vaccinated mice complete protection against challenge with the heterologous genotype SD12(GI) strain. The protection rates against SD12(GI) strain challenge were 80% (Fig 2A), 60% (Fig 2B), and 40% (Fig 2C) in the groups vaccinated with high, medium and low doses, respectively. These data indicated a partial protective efficacy of the SA14-14-2 vaccine against the heterologous genotype SD12(GI) strain.

### Reduced protective efficacy of N28(GIII)-inactivated vaccine against GI strain challenge in mice

**Fig 3:**
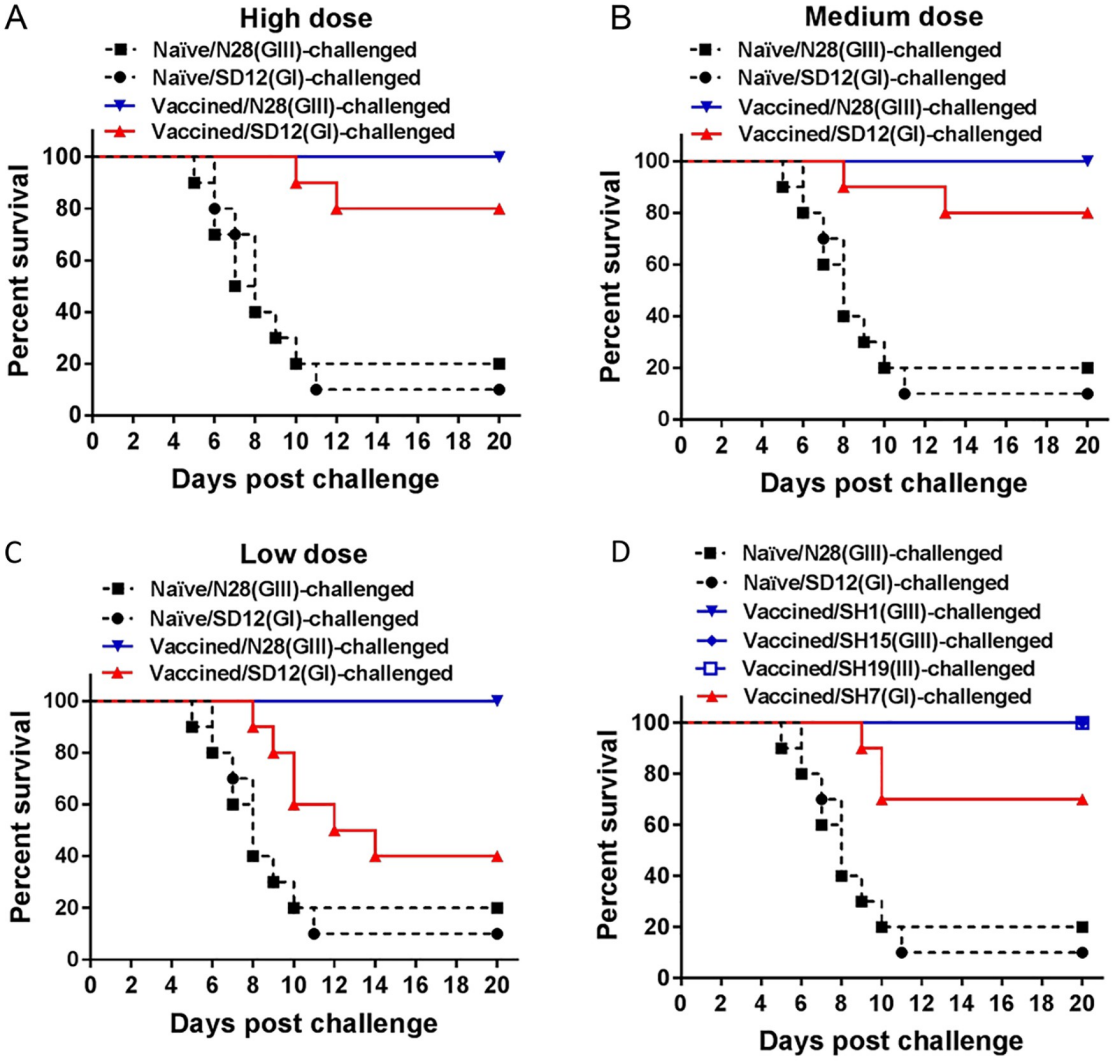
Protective efficacy of a N28(GIII)-inactivated vaccine. Mice (*n* = 10) were mock-vaccinated (Naïve) or immunized with N28(GIII)-inactivated vaccine at high (10^5^ PFU) dose (A), at medium (10^4^ PFU) dose (B and D), and at low (10^3^ PFU) dose (C) and challenged with the indicated JEV strains.

To confirm the reduced protective efficacy of the GIII-derived vaccine against GI viral infection, mice were immunized with N28(GIII)-inactivated vaccine at high (10^5^ PFU), medium (10^4^ PFU), or low (10^3^ PFU) doses and challenged with the homologous N28(GIII) and heterologous genotype SD12(GI) strains, respectively. N28(GIII)-inactivated vaccine protected the vaccinated mice completely at high, medium and low doses against the homologous N28(GIII) strain (Fig 3A, 3B and 3C). However, the protection rates against the heterologous genotype SD12(GI) strain challenge were 80% (Fig 3A), 80% (Fig 3B) and 40% (Fig 3C) in the groups vaccinated with high, medium and low doses, respectively. This suggested a reduced protective efficacy against the heterologous genotype SD12(GI) strain.

The protective efficacy of the N28(GIII)-inactivated vaccine were further examined at a medium dose against other homologous genotype viruses including SH1(GIII), SH15(GIII) and SH19(GIII) strains, and the heterologous genotype SH7(GI) strain. N28(GIII)-inactivated vaccine protected the vaccinated mice completely against the challenges of the homologous genotype SH1(GIII), SH15(GIII) and SH19(GIII) strains, but not against the challenge of the heterologous genotype SH7(GI) strain (Fig 3D). The protection rate was 70% in the group challenged with SH7(GI) strain. Taken together, these data support the finding of reduced protective efficacy of N28(GIII)-inactivated vaccines against heterologous genotype GI strains.

### Reduced protective efficacy of SD12(GI)-inactivated vaccine against GIII strain challenge in mice

**Fig 4:**
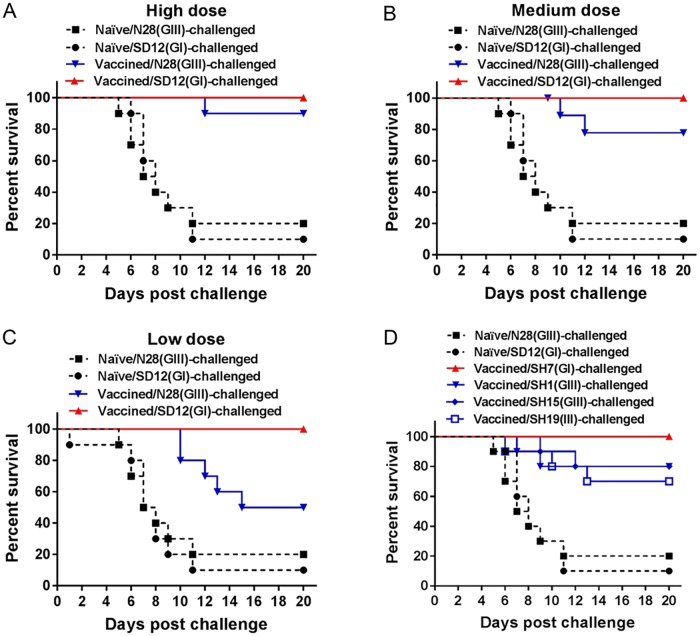
Protective efficacy of a SD12(GI)-inactivated vaccine. Mice (*n* = 10) were mock-vaccinated (Naïve) or immunized with SD12(GI)-inactivated vaccine at high (10^5^ PFU) dose (A), medium (10^4^ PFU) dose (B and D) and low (10^3^ PFU) dose (C), and challenged with the indicated JEV strains.

Given the emergence of the GI virus as the dominant genotype in Asian regions and the partial cross-protection efficacy of GIII-derived vaccines against GI viral infection, development of a GI-derived vaccine has been proposed to control GI viral infection [25,28,29]. We therefore examined the protective efficacy of a GI-derived vaccine against the challenge of GI and GIII viruses. Mice were vaccinated with SD12(GI)-inactivated vaccine at high (10^5^ PFU), medium (10^4^ PFU), or low (10^3^ PFU) doses and challenged with the homologous SD12(GI) and heterologous genotype N28(GIII) strains, respectively. SD12(GI)-inactivated vaccine protected the vaccinated mice completely against the homologous SD12(GI) strain, but not against the heterologous genotype N28(GIII) strain (Fig 4). The protection rates against N28(GIII) strain challenge were 90% (Fig 4A), 80% (Fig 4B) and 50% (Fig 4C) in the groups vaccinated with high, medium and low doses, respectively, suggesting a reduced protective efficacy against the heterologous genotype N28(GIII) strain.

The protective efficacy of SD12(GI)-inactivated vaccine were further examined at a medium dose against another homologous genotype strain SH7(GI) and heterologous genotype viruses including SH1(GIII), SH15(GIII) and SH19(GIII) strains. SD12(GI)-inactivated vaccine protected the vaccinated mice completely against the homologous genotype SH7(GI) strain, but not against the challenge of heterologous genotype SH1(GIII), SH15(GIII) and SH19(GIII) strains (Fig 4D). The protection rate was 80%, 80% and 70% in the group challenged with SH1(GIII), SH15(GIII) and SH19(GIII), respectively. Taken together, these data indicated the reduced protective efficacy of SD12(GI)-inactivated vaccine against the heterologous genotype GIII strains.

## Discussion

The emergence of GI JEV as the dominant genotype in Asian regions [6,7] has raised concerns about the efficacy of GIII-derived vaccines against GI viral infection [13]. The goal of this study was to examine the cross-protection between GI and GIII viruses to estimate the potential need for new vaccine strategies for control of both GI and GIII viral infection.

Neutralizing antibodies play the most important role in protection of JEV infection and the neutralizing antibody titer correlates with this protection [30,31]. We therefore measured the neutralizing antibody titers in pigs and mice vaccinated with the SA14-14-2 vaccine and observed that the neutralizing antibody titers specific to the heterologous genotype SD12(GI) strain were significantly lower than those specific to the homologous SA14-14-2 strain and homologous genotype N28(GIII) strain. These results were consistent with previous observations that the neutralizing antibody titers specific to GI viruses were lower than those specific to GIII viruses in humans and pigs vaccinated with GIII-derived vaccines [18, 21, 32–34].

The SA14-14-2 vaccine is currently used for control of JE in both humans and pigs [12]. We therefore evaluated the protective efficacy of the SA14-14-2 vaccine against GI challenge using a mouse challenge model. The SA14-14-2 vaccine provided vaccinated mice with complete protection against the homologous genotype N28(GIII) strain, but not against the heterologous genotype SD12(GI) strain. The protection rates against the heterologous genotype SD12(GI) strain were 60%–80%, suggesting that vaccination was partially protective when using a GIII-derived vaccine. This observation was inconsistent with a previous observation that the SA14-14-2 vaccine affords mice similar protection against both GI and GIII strain challenge [26], in which KM strain mice immunized with the SA14-14-2 vaccine had an 80%–100% protection rate against the both GI and GIII strains. This apparent discrepancy between our and Liu *et al*.’s results may be attributable to differences in the mouse strains used for the JEV challenge. The mouse strain used in Liu *et al*.’s study was the KM strain [26], whereas the mouse strain used in our study was C57BL/6. We have observed that C57BL/6 strain mice were more sensitive to JEV challenge than KM strain mice. This explanation is also supported by differences in the neutralizing antibody titers. Our and several previous observations demonstrated the reduced neutralizing antibody titers specific to GI viruses in individuals immunized with GIII-derived vaccines [18,21,34]. However, the reduced neutralizing antibody titers specific to GI viruses were not observed in KM strain mice vaccinated with the SA14-14-2 vaccine in Liu *et al*.’s study [26]. In addition, we challenged the vaccinated mice at 14 days post-vaccination and observed the reduced protection against the heterologous genotype strains. The protective efficacy might be changed if the vaccinated mice were challenged at different interval days post-vaccination.

The difference in susceptibility to flavivirus infection between C57BL/6 and KM mice has also been observed in infection by Zika virus that is related to JEV in the family *Flaviviridae*. C57BL/6 mice after Zika virus infection showed higher morbidity (100%) than KM mice (62%) [35]. The specific reasons for the different susceptibility between these two mouse strains are unknown at present, probably attributable to their genetic difference. KM mouse with white coat color is outbred strain originated from the Swiss mice, while C57BL/6 mouse with dark brown coat color is inbred strain derived from the C57BL mice.

To confirm the reduced protective efficacy of GIII-derived vaccines against GI viral infection, we immunized mice with N28(GIII)-inactivated vaccine and challenged with the heterologous genotype GI strains. The reason we used the newly-isolated N28(GIII) strain rather than the existing licensed inactivated JE vaccine was that the inactivated GIII Beijing-3 (P3) vaccine available in China is licensed 20 years ago. Several amino acid variations have been observed in E protein between the inactivated Beijing-3 (P3) vaccine and the recently-isolated strains [36]. N28(GIII)-inactivated vaccine provided the vaccinated mice complete protection against the challenge of homologous genotype GIII strains, but not against the challenge of heterologous genotype GI strains, confirming the reduced protective efficacy of GIII-derived vaccines against GI viral infection. These results supported the concern of increased risk of GI infection in populations immunized with GIII-derived vaccines. Indeed, GI virus has been isolated from patient vaccinated with the SA14-14-2 vaccine in China [22]. A similar case was also reported in India [19]. These epidemiological data together with the reduced protective efficacy of GIII-derived vaccines against GI virus infection suggest a potential need for a GI-derived vaccine. In fact, several trials for the development of a GI-derived vaccine have been reported [25,28,29].

Although GI has replaced GIII as the dominant genotype, the co-circulation of GI and GIII viruses is present in many Asian countries [8–10]. We therefore examined the protective efficacy of a GI-derived vaccine against GIII viral infection. Mice were immunized with SD12(GI)-inactivated vaccine and challenged with heterologous genotype GIII strains. SD12(GI)-inactivated vaccine provided the vaccinated mice complete protection against the challenge of homologous genotype GI strains, but not against the challenge of heterologous genotype GIII strains. This suggests a partial protective efficacy of GI-derived vaccines against GIII viral infection. These results were consistent with a previous observation that GI virus-like particles failed to cross-protect 10% of vaccinated mice against GIII viral challenge [29]. However, swine immunized with these GI virus-like particles showed no fever, viremia or viral RNA in tissues after GI or GIII viral challenge, demonstrating sterile protection against GI and GIII viral infection in swine [29]. Therefore, more detailed studies are needed to evaluate the cross-protective efficacy of GI-derived vaccines against GIII viral infection.

In conclusion, the partial cross-protective efficacy of JE vaccines against heterologous genotype JEV challenge was observed using a mouse challenge model. GIII-derived vaccines failed to provide the vaccinated mice complete protection against the challenge of heterologous genotype GI strains. Additionally, vaccination of mice with a GI-inactivated vaccine conferred only partial protection against the challenge of heterologous genotype GIII strains. The partial cross-protection between GI and GIII viruses suggest a potential need for new JE vaccine strategies, including options like a bivalent vaccine, to control both genotype infection. However, the current data obtained from a mouse challenge model cannot be simply extrapolated to the vaccination strategy for humans, more comprehensive studies should be conducted to address the partial cross-protective efficacy of JE vaccines against the heterologous genotype using JEV natural hosts.

## Supporting information

S1 Fig Detection of neutralizing antibody titers (PRNT_90_) in pigs and mice vaccinated with the SA14-14-2 vaccine. (A and B) Pigs (*n* = 40) were immunized with SA14-14-2 vaccine and serum samples for each animal were collected at 30 days post-vaccination for detection of neutralizing antibody titers (PRNT_90_) against SA14-14-2, N28(GIII) and SD12(GI) strains. (A) Seropositive rate against the indicated JEV strains. A *p* value was generated by Fisher’s exact test. (B) PRNT_90_ against the indicated JEV strains were measured on BHK-21 cells and plotted. A *p* value was generated by Student’s *t*-test. (C, D and E) Mice (*n* = 10) were immunized with the SA14-14-2 vaccine and serum samples were collected at 14 days post-vaccination for detection of PRNT_90_ against SA14-14-2 vaccine, N28(GIII) and SD12(GI) strains. (C) PRNT_90_ titers in serum samples collected from pre-vaccinated mice. (D) PRNT_90_ titers against the indicated JEV strains in serum samples collected from vaccinated mice. A *p* value was generated by Student’s *t*-test. (E) Seropositive rate against the indicated JEV strains. A *p* value was generated by Fisher’s exact test.
(TIF)

S1 Table Information of JEV strains. (DOCX)
