# Supplementary figures and images for: Partial cross-protection between Japanese encephalitis virus genotype I and III in mice
Source: PLoS Negl Trop Dis. 2019 Aug 2;13(8):e0007601. doi: 10.1371/journal.pntd.0007601 (PMC6693775; doi:10.1371/journal.pntd.0007601)

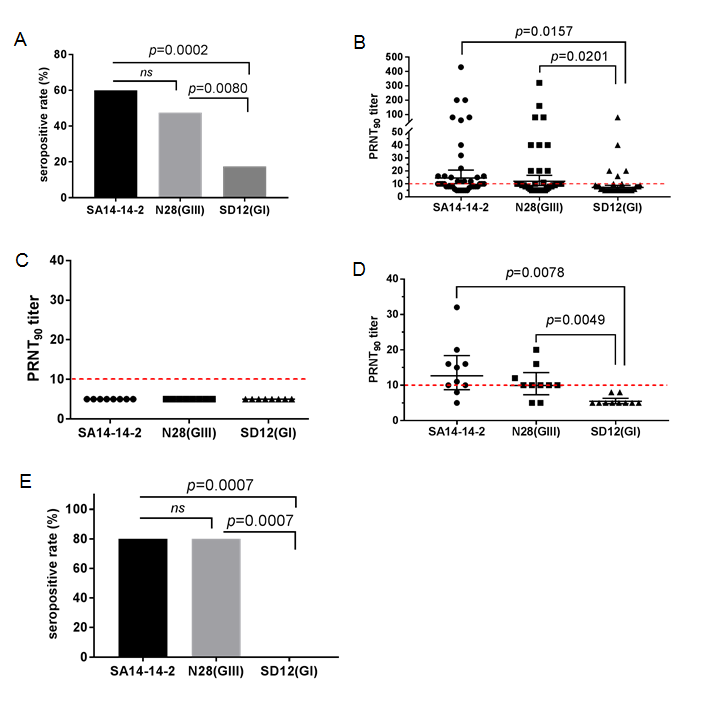

Supplement: S1 Fig — (A and B) Pigs (n = 40) were immunized with SA14-14-2 vaccine and serum samples for each animal were collected at 30 days post-vaccination for detection of neutralizing antibody titers (PRNT90) against SA14-14-2, N28(GIII) and SD12(GI) strains. (A) Seropositive rate against the indicated JEV strains. A p value was generated by Fisher’s exact test. (B) PRNT90 against the indicated JEV strains were measured on BHK-21 cells and plotted. A p value was generated by Student’s t-test. (C, D and E) Mice (n = 10) were immunized with the SA14-14-2 vaccine and serum samples were collected at 14 days post-vaccination for detection of PRNT90 against SA14-14-2 vaccine, N28(GIII) and SD12(GI) strains. (C) PRNT90 titers in serum samples collected from pre-vaccinated mice. (D) PRNT90 titers against the indicated JEV strains in serum samples collected from vaccinated mice. A p value was generated by Student’s t-test. (E) Seropositive rate against the indicated JEV strains. A p value was generated by Fisher’s exact test. (TIF) [file pntd.0007601.s001.tif]
