# Supplementary material for: Partial cross-protection between Japanese encephalitis virus genotype I and III in mice
Source: PLoS Negl Trop Dis. 2019 Aug 2;13(8):e0007601. doi: 10.1371/journal.pntd.0007601 (PMC6693775; doi:10.1371/journal.pntd.0007601)
Supplement: S1 Table — (DOCX) [file pntd.0007601.s002.docx]

**S1 Table. Information of JEV strains**

| Strains | Genotype | GenBank No. | 50% lethal dose (LD_50_)* |
| --- | --- | --- | --- |
| SA14-14-2 | III | AF315119 | N/A |
| N28 | III | MH753126 | 10^3.00^ |
| SH1 | III | MH753128 | 10^5.00^ |
| SH15 | III | MH753130 | 10^4.31^ |
| SH19 | III | MH753131 | 10^3.84^ |
| SD12 | I | MH753127 | 10^2.70^ |
| SH7 | I | MH753129 | 10^5.10^ |

*, 50% lethal dose (LD_50_) of each JEV strain was tested on 3-week-old C57BL/6 strain mice by intraperitoneal inoculation of serially diluted JEV.
